# Can pattern and depth of invasion predict lymph node relapse and prognosis in tongue squamous cell carcinoma

**DOI:** 10.1186/s12885-019-5859-y

**Published:** 2019-07-19

**Authors:** Kailiu Wu, Junshui Wei, Zhengwu Liu, Binbin Yu, Xi Yang, Chunye Zhang, Ahmed Abdelrehem, Chenping Zhang, Siyi Li

**Affiliations:** 1 Shanghai Key Laboratory of Stomatology, Department of Oral and Maxillofacial-Head and Neck Oncology, Ninth People’s Hospital, School of Stomatology, Shanghai Jiao Tong University School of Medicine, Shanghai, 200011 China; 2 Department of Oral and Maxillofacial-Head and Neck Oncology, Fengcheng Hospital & Shanghai Ninth People’s Hospital (Fengcheng Branch Hospital) Affiliated to School of Medicine of Shanghai Jiao Tong University, Shanghai, 201411 China; 3 Department of Stomatology, Taizhou First People’s Hospital, Taizhou, 318020 China; 4 Department of Oral and Maxillofacial Surgery, Third Attached Hospital, Guangxi Medical University, Nanning, 530031 China; 5 Shanghai Key Laboratory of Stomatology, Department of oral pathology, Ninth People’s Hospital, School of Stomatology, Shanghai Jiao Tong University School of Medicine, Shanghai, 200011 China; 6 Lecturer, Department of Craniomaxillofacial and Plastic Surgery, Faculty of Dentistry, Alexandria University, Alexandria, Egypt

**Keywords:** Elective neck dissection, Head and neck cancer, Invasive front, Survival analysis, Prognosis, Cohort studies

## Abstract

**Background:**

Tongue squamous cell carcinoma (TSCC) is a special type of oral cancer. Cervical lymph node relapse may occur in a large percentage of TSCC patients, which usually indicates poor prognosis. In this cohort study, we focused on the predictive value of the pathological features on cervical lymph node relapse and TSCC prognosis (disease free survival).

**Methods:**

One hundred forty-one TSCC patients staged as T_1–2_N_0_ were enrolled and categorized. Subjects were followed-up for 60 months. Univariate analysis was performed with Chi-square test for cervical lymph node relapse and Kaplan-Meier survival analysis and log rank *P* value for patient prognosis; multivariate analysis was also utilized with Cox regression.

**Results:**

In univariate analysis, trabes growth pattern, depth of invasion greater than 4 mm, poor pathological differentiation and neurovascular invasion were considered as risk factors for cervical lymph node relapse and poor prognosis. In multivariate analysis, only patients with trabes growth pattern in the invasive front or depth of invasion larger than 4 mm had a higher risk of metastasis. Elder age group and trabes growth pattern of invasive front were considered as predictors of poor prognosis. Bad habits of smoking and alcohol consumption were related to the higher risk of metastasis.

**Conclusion:**

Trabes growth pattern of invasive front was a potent risk factor for TSCC cervical lymph node relapse and indicated poor prognosis. Preventive therapy including selective neck dissection was thus suggested for certain patients.

**Trial registration:**

Not applicable.

## Background

Globally, oral cancer is a huge threat to our health. Approximately 300,400 people were diagnosed and 145,400 died of oral cancer in a year [1]. Among all types of oral cancer, squamous cell carcinoma (SCC) accounts for more than 95%. Derived from stratified squamous epithelium of the oral mucosa, SCC primarily occurred at the buccal mucosa and tongue [2]. Tobacco, alcohol use, radiation and human papillary virus infection are implicated as risk factors for SCC [3].

Tongue squamous cell carcinoma (TSCC) is a special type of oral cancer. Patients may present with non-healing ulcers and pain in the mouth. TSCC usually grows as infiltrative and/or exophytic lesions and presents with local invasion. Dysarthria during the development of the disease implies the invasion of the tumor into the deep muscles. TSCC do not often have distant metastasis, but cervical lymph node relapse may occur in a large percentage of TSCC patients, which usually indicates poor prognosis. Analysis concerning the risk factors for cervical lymph node relapse is thus critical for clarifying the onset and progression of TSCC as well as improving the prognosis of patients. Few studies have been focusing on the clinical and pathological factors related to the prognosis of TSCC [4]. Yet few reports investigated the pathological features of TSCC.

It was established that tumor cells at the invasive front differed substantially from the central and superficial tumor cells. The invasive front of tumor often indicated the aggressiveness of TSCC [5]. In addition, the depth of invasion may also reflect the progression of tumor. Moreover, it was reported in esophageal tumors that the invasive front acted as predictive factors for metastasis and prognosis [6]. In this cohort study, using both univariate and multivariate analyses, we focused on the predictive value of the pathological features on cervical lymph node relapse and TSCC prognosis.

## Methods

### Subjects

Subjects involved in this study were recruited from the outpatient clinic at Shanghai Jiao Tong University School of Medicine. All patients were pathologically confirmed as TSCC by having biopsy before surgery and then staged as T_1–2_ with no cervical lymph node relapse (N_0_). The TNM stage was evaluated by the clinical check and with the help of CT and B-ultrasound. TSCC samples were collected following tumor resection. No radiotherapy or chemotherapy had been performed before the collection of tumor samples. Baseline information including gender, age, pathological features of tumor, location of tumor (anterior, middle and posterior), habits of alcohol consumption and smoking, and treatment (neck dissection vs. observation) was documented. Pathological features of TSCC samples included the growth pattern of the invasive front (crumb vs. trabes), depth of invasion (< 4 mm vs. ≥4 mm), grade of pathological differentiation (well vs. Moderate/poor), and neurovascular invasion (yes vs. no). The growth pattern of the invasive front was determined by the pathologists at the hospital according to Rivera et al. [7]. This study was performed following the protocols approved by the Ethical Committee of Shanghai Jiao Tong University School of Medicine. An informed consent was signed by all participants in this clinical study.

### Therapy

Patients enrolled in the current study were randomly divided into neck dissection group and observation group. An elective supraomohyoid neck dissection was performed for patients in the neck dissection group. In cases with TSCC recurrence and cervical lymph node relapse during the follow-up, a corresponding therapy was applied to patients according to their condition. Radical resection, chemotherapy and radiotherapy might be included.

### Follow-up

The maximum follow-up duration was 60 months. During the follow-up period, patients were examined for tumor recurrence and cervical lymph node relapse every 3 months during the first 24 months, then every 6 months for the following 36 months. Examination included clinical inspection and CT or MRI. All nodal relapse patients were pathologically proven by surgical pathology.

### Data analysis

In this study, we focused on the association between the clinicopathological features of TSCC and patient prognosis (disease free survival), in addition to collecting both the Disease specific survival (DSS) and the Disease free survival (DFS). Univariate analysis was performed with Chi-square test for cervical lymph node relapse and Kaplan-Meier survival analysis and log rank *P* value for patient prognosis; multivariate analysis was performed with Cox regression. Variations including gender, age, pathological features of tumor, location of tumor, habits of drinking and smoking, and treatment were involved in the growth pattern. Odd ratio (OR), hazard ratio (HR) and their corresponding 95% confidence interval (CI) were calculated to measure the association. SPSS software, version 17.0 (SPSS, Chicago, IL) was used for the analysis, with *P* <  0.05 being significant.

## Results

### Characteristics of subjects

**Fig. 1:**
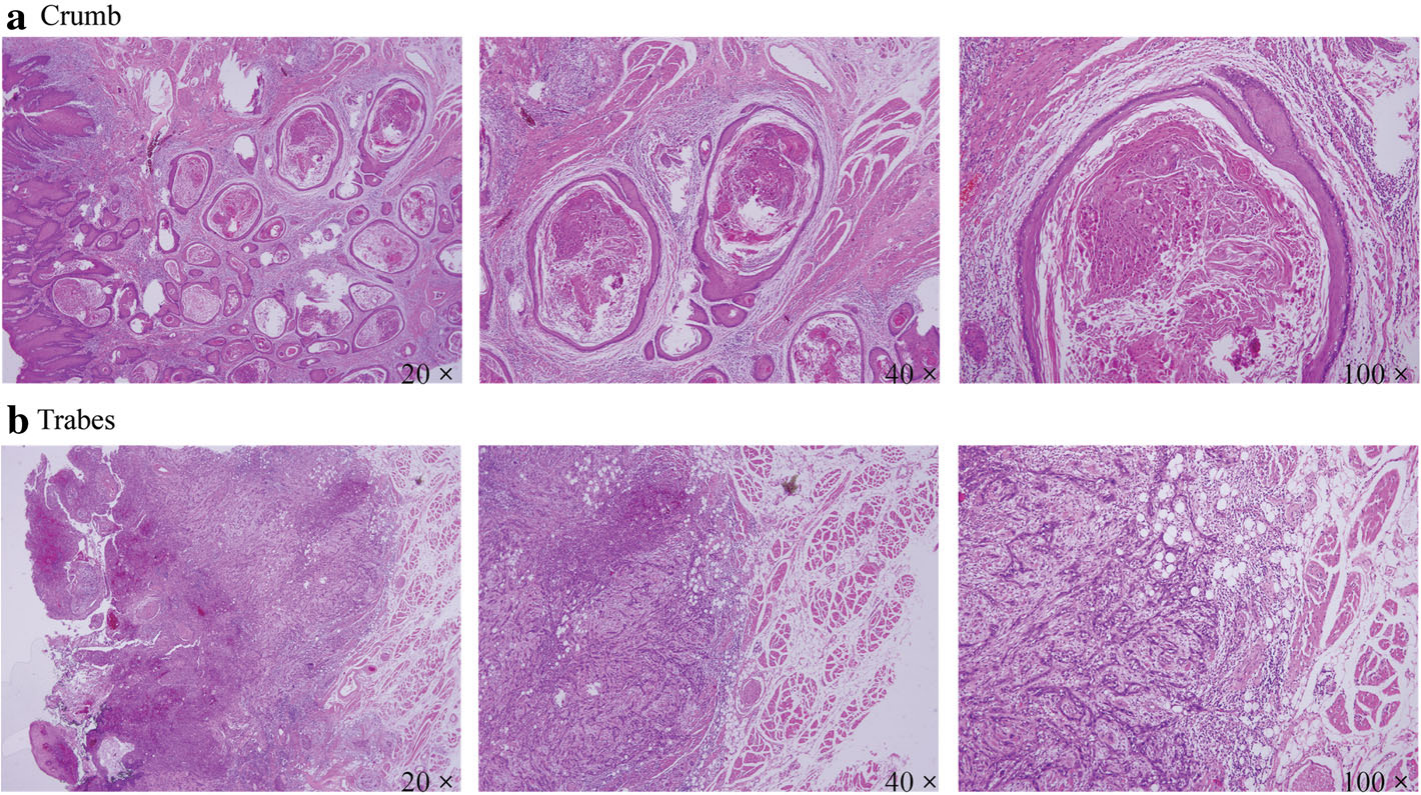
Different pathological features of TSCC, crumb (islet-infiltrating cells with wide fronts of invasion) and trabes (thin infiltrating cords). **a** represents that the growth pattern of the invasion front invades the surrounding tissue like a rope, indicating a poor prognosis. **b** represents that the growth pattern of the invasion front advances like a clump forward, indicating a better prognosis relative to **a**

This is a cohort study concerning the association between clinicopathological features of tumor and TSCC prognosis. A total of 141 TSCC patients (75 males and 66 females) were enrolled in this study. The average age of patients was 55 years. Majority of tumors (*n* = 103) were located in the middle part of the tongue. Sixty patients were at T1 stage and 81 at T2. Neck dissection was performed in 69 patients, while 72 patients were categorized in the observation group. Concerning pathological features of TSCC, samples were categorized into crumb (islet-infiltrating cells with wide margins of invasion) and trabes (thin infiltrating cords), as presented in Fig. 1. Seventy nine patients had an invasive front in the crumb pattern, while 62 in the trabes pattern. Ninety eight patients had a depth of invasion greater than 4 mm. Neurovascular invasion was observed in 18 patients and only 48 patients have well differentiated tumors. Furthermore, 59 patients had the habit of smoking and 52 had the habit of drinking. The cervical lymph node relapse was observed in 36 patients. The average follow-up duration was 37 months. The results were summarized in Table 1. Patients with cervical lymph node relapse showed significantly altered pathological features including growth pattern of invasive front, depth of invasion, grade of pathological differentiation, and neurovascular invasion (*P* <  0.05). Moreover, location of the tumor, T stage, habit of drinking and smoking influenced the metastasis of TSCC (*P* <  0.05). However, patients with and without neck dissection had similar risk of cervical lymph node relapse (*P* = 0.191).

**Table 1:** Baseline characteristics of the included tongue squamous cell carcinoma patients

Items	Cases (*n* = 141)	Metastases (+)	Metastases (−)	*P* value^*^
Gender				
Male	75	24	51	0.061
Female	66	12	54	
Age (years old)				
< 55	59	14	45	0.678
≥ 55	82	22	60	
Growth mode of invasive front				
Crumb	79	8	71	< 0.001
Trabes	62	28	34	
Depth of invasion				
< 4 mm	43	1	42	< 0.001
≥ 4 mm	98	35	63	
Pathological differentiation				
Well	48	7	41	0.032
Moderate/poor	93	29	64	
Neurovascular invasion				
Yes	18	11	7	< 0.001
No	123	25	98	
Location				
Anterior	14	1	13	0.053
Middle	103	25	78	
Back	24	10	14	
T stage				
T1	60	8	52	0.004
T2	81	28	53	
Smoking				
Yes	59	21	38	0.02
No	82	15	67	
Drinking				
Yes	52	21	31	0.002
No	89	15	74	
Treatment				
Neck dissection	69	21	48	0.191
Observation	72	15	57	

P^*^: chi-square test

### Clinical features and lymph node relapse

Subsequently, we investigated the association between clinicopathological features and cervical lymph node relapse with OR and corresponding 95% CI. As illustrated in Table 2, trabes growth pattern was related to a higher risk of lymph node relapse (OR = 7.31, 95%CI: 3.01–17.72). Depth of invasion larger than 4 mm, pattern of invasive front, poor pathological differentiation and neurovascular invasion were all considered as risk factors for cervical lymph node relapse (OR = 23.33, OR = 2.65, OR = 6.16). Furthermore, TSCC in the posterior region of the tongue indicated a high risk of metastasis as well (OR = 9.29, 95%CI: 1.04–82.96). Moreover, Bad living habits including smoking and drinking increased the risk of cervical lymph node relapse (OR = 2.47, OR = 3.34). Neck dissection did not significantly reduce the risk of metastasis (OR = 1.66, 95%CI: 0.77–3.58).

**Table 2:** Univariate and multivariate analyses about parameters relevant to lymph node relapse

Items	All patients (n)	With lymph node relapse	Without lymph node relapse	Univariate analysis		Multi-variate analysis	
				OR (95%CI)	*P* value*	OR (95%CI)	*P* value#
Gender							
Male	75	24	51	Reference		Reference	
Female	66	12	54	0.47 (0.21–1.04)	0.063	0.64 (0.15–2.69)	0.541
Age (years old)							
< 55	59	14	45	Reference		Reference	
≥ 55	82	22	60	1.18 (0.54–2.56)	0.677	1.23 (0.45–3.34)	0.683
Growth mode of invasive front							
Crumb	79	8	71	Reference		Reference	
Trabes	62	28	34	7.31 (3.01–17.72)	**0.001**	5.03 (1.52–16.68)	**0.008**
Depth of invasion							
< 4 mm	43	1	42	Reference		Reference	
≥ 4 mm	98	35	63	23.33 (3.08–176.91)	**0.002**	12.23 (1.31–113.90)	**0.028**
Pathological differentiation							
Well	48	7	41	Reference		Reference	
Moderate/poor	93	29	64	2.65 (1.06–6.62)	**0.036**	0.76 (0.22–2.62)	0.667
Neurovascular invasion							
No	123	25	98	Reference		Reference	
Yes	18	11	7	6.16 (2.17–17.51)	**0.001**	2.20 (0.61–7.97)	0.232
Location							
Anterior	14	1	13	Reference		Reference	
Middle	103	25	78	4.17 (0.52–33.46)	0.179	5.05 (0.52–49.44)	0.164
Back	24	10	14	9.29 (1.04–82.96)	**0.046**	5.74 (0.50–66.52)	0.162
T stage							
T1	60	8	52	Reference		Reference	
T2	81	28	53	3.43 (1.43–8.23)	**0.006**	1.42 (0.46–4.35)	0.543
Smoking							
No	82	15	67	Reference		Reference	
Yes	59	21	38	2.47 (1.14–5.35)	**0.022**	0.05 (0.00–2.00)	0.11
Drinking							
No	89	15	74	Reference		Reference	
Yes	52	21	31	3.34 (1.53–7.32)	**0.003**	33.62 (0.97–1169.50)	0.052
Treatment							
Observation	72	15	57	Reference		Reference	
Neck dissection	69	21	48	1.66 (0.77–3.58)	0.193	1.15 (0.41–3.26)	0.794

*OR* odds ratio, *CI* confidence interval; P*: uni-variate regression analysis; *P*#: multi-variate regression analysis. Note: entries in boldface means: *P* < 0.05

However, in multivariate analysis, only growth pattern of invasive front and depth of invasion were observed to have significant effects on the status of cervical lymph node relapse. Patients with trabes growth pattern in the invasive front and depth of invasion larger than 4 mm had a higher risk of metastasis (OR = 5.03, 95%CI: 1.52–16.68; OR = 12.23, 95%CI: 1.31–113.90).

### Clinical features and TSCC prognosis

**Fig. 2:**
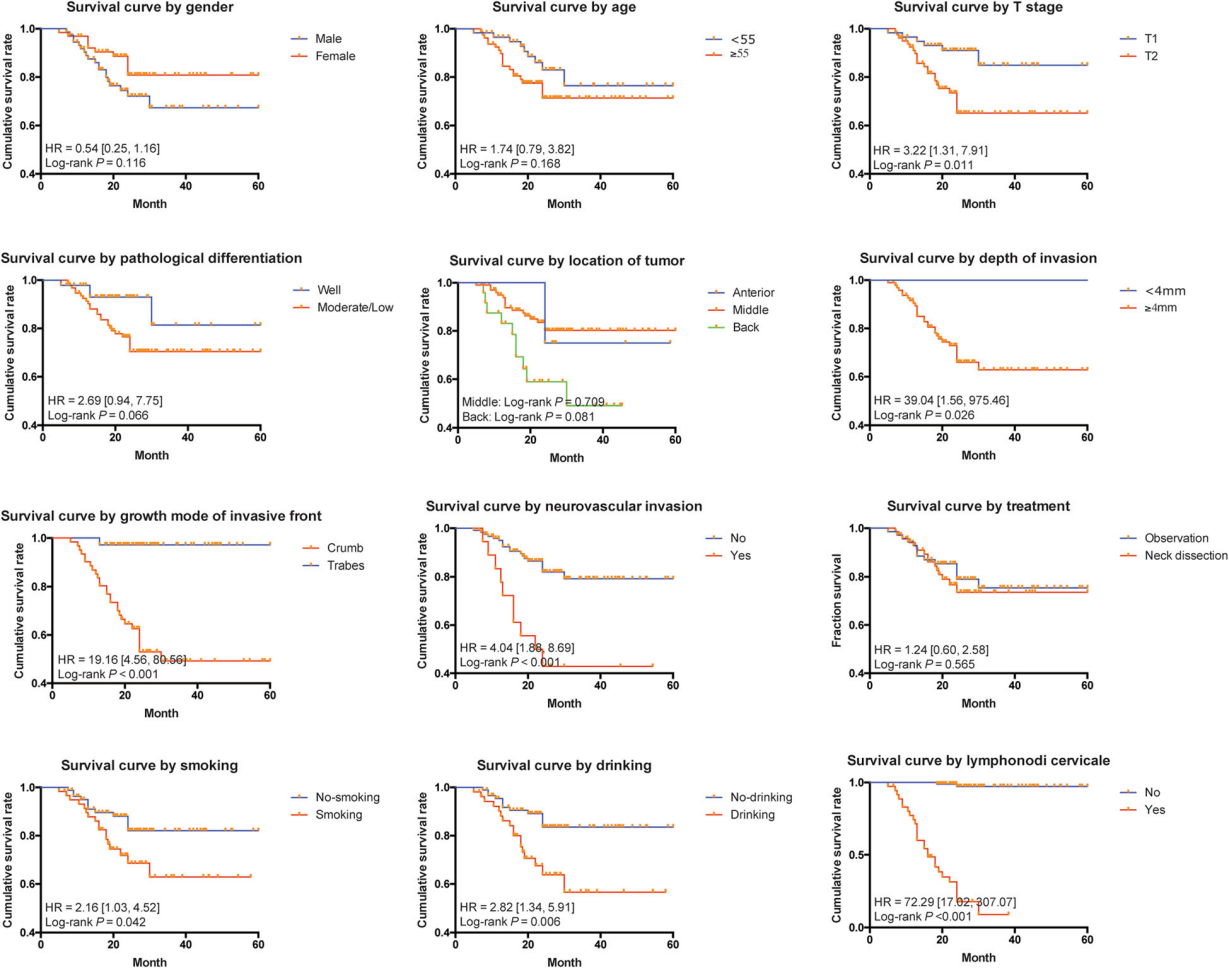
Survival curve for SCC patients with different clinical characteristic

Considering the effect of clinicopathological features on TSCC prognosis, both univariate and multivariate analyses were performed. In Kaplan-Meier survival analysis, we found that the growth pattern of invasive front, depth of invasion, neurovascular invasion, location of the tumor, T stage, habit of smoking and drinking could significantly influence the prognosis of TSCC patients, as illustrated in Fig. 2 (log rank *P* <  0.05). The effect was measured with HR and corresponding 95%CI as presented in Table 3. Cervical lymph node relapse also predicted poor prognosis (HR = 72.29, 95%CI: 17.02–307.07). However, according to our results, neck dissection did not help to improve the prognosis of TSCC patients significantly (HR = 1.24, 95%CI: 0.60–2.58, *P* = 0.565).

**Table 3:** Univariate and multivariate analyses on factors associated with TSCC prognosis

Items	N	Univariate analysis			Multi-variate analysis		
		HR	95%CI	*P* value^*^	HR	95%CI	*P* value^#^
Gender							
Male	75	Reference			Reference		
Female	66	0.54	0.25–1.16	0.116	0.33	0.06–1.79	0.201
Age (years old)							
< 55	59	Reference			Reference		
≥ 55	82	1.74	0.79–3.82	0.168	3.9	1.29–11.82	**0.016**
Growth mode of invasive front							
Crumb	79	Reference			Reference		
Trabes	62	19.16	4.56–80.56	**< 0.001**	6.3	1.15–34.43	**0.034**
Depth of invasion							
< 4 mm	43	Reference			Reference		
≥ 4 mm	98	39.04	1.56–975.46	**0.026**	237,144.33	0.00–8.820E+ 118	0.926
Pathological differentiation							
Well	48	Reference			Reference		
Moderate/poor	93	2.69	0.94–7.75	0.066	0.913	0.20–4.09	0.905
Neurovascular invasion							
No	123	Reference			Reference		
Yes	18	4.04	1.88–8.69	**< 0.001**	0.48	0.19–1.24	0.129
Location							
Anterior	14	Reference			Reference		
Middle	103	1.32	0.31–5.73	0.709	2.51	0.45–14.03	0.296
Back	24	3.87	0.85–17.68	0.081	4.66	0.62–35.02	0.135
T stage							
T1	60	Reference			Reference		
T2	81	3.22	1.31–7.91	**0.011**	1.23	0.35–4.32	0.752
Smoking							
No	82	Reference			Reference		
Yes	59	2.16	1.03–4.52	**0.042**	0.00	0.00–2.917E+ 078	0.929
Drinking							
No	89	Reference			Reference		
Yes	52	2.82	1.34–5.91	**0.006**	2162.96	0.00–3.651E+ 085	0.937
Treatment							
Observation	72	Reference			Reference		
Neck dissection	69	1.24	0.60–2.58	0.565	0.96	0.40–2.30	0.922
Lymphonodi cervicale							
No	105	Reference			Reference		
Yes	36	72.29	17.02–307.07	< 0.001	36.7	7.68–175.35	< 0.001

*HR* hazard ratio, *CI* confidence interval; P^*^: uni-variate regression analysis; *P*^#^: multi-variate regression analysis. Note: entries in boldface means: *P* < 0.05

In the multivariate analysis, the effect of clinical features on TSCC prognosis was analyzed with Cox regression. Age elder than 55 and trabes pattern of invasion were considered as predictors of poor prognosis (HR = 3.9, 95%CI: 1.29–11.82; HR = 6.3, 95%CI: 1.15–34.43). Cervical lymph node relapse independent of other variations was also considered as a potent indicator for poor prognosis (HR = 36.7; 95%CI: 7.68–175.35).

According to our results, there was a strong correlation between the trabes growth pattern and DOI as illustrated in Table 4.

**Table 4:** Chi-square test analyses on pattern and DOI

Pattern	DOI		Lymph Node	Chi-square test	
	< 4 mm	> = 4 mm		OR (95%CI)	*P* Value
Crumb	34	37	Relapse(−)	2.986 (1.191–7.487)	0.017
Trabes	8	26			
Crumb	2	14	Relapse(+)	11.4 (1.105–117.7)	0.013
Trabes	0	56			
Crumb	36	51	Total	7.235 (3.117–16.794)	< 0.001
Trabes	8	82			

## Discussion

The present study investigated the correlation between the clinicopathological features, cervical lymph node relapse and TSCC prognosis. Both univariate and multivariate analyses were performed. In the univariate analysis, trabes growth pattern, depth of invasion larger than 4 mm, pattern of invasive front, poor pathological differentiation and neurovascular invasion were all considered as risk factors for cervical lymph node relapse. TSCC in the posterior tongue region and unfavorable habits of smoking and drinking were related to the higher risk of metastasis. However, in the multivariate analysis, only patients with trabes growth pattern in the invasive front and depth of invasion larger than 4 mm were found to have higher risk of metastasis. Similarly, concerning the prognosis of TSCC patients, in univariate analysis, trabes growth pattern of invasive front, depth of invasion larger than 4 mm, neurovascular invasion, habits of smoking and drinking were associated with poor TSCC prognosis. In multivariate analysis, only elder age and trabes growth pattern of invasive front were considered as predictors of poor prognosis.

As previously reported, the average age of oral cancer was 63 years old [8]. In addition, elderly males usually suffered a higher risk of TSCC. In the current study, we observed that age was not significantly associated with cervical lymph node relapse both in univariate and multivariate analyses. However, age may serve as a critical predictor for the prognosis of TSCC. Old patients diagnosed at an age elder than 55 had significantly poorer prognosis compared with younger group.

Growth pattern of the tumor, depth of invasion, pathological differentiation and neurovascular invasion all indicated the aggressiveness of the tumor. Here in the univariate analysis, they were all proved to be risk factors for TSCC metastasis. However, some of such factors might be dependent on others. Hence a multivariate analysis was performed to clarify the independent risk factors. In the cox regression analysis, adjusted by gender, age, pathological features of tumor, location of tumor and treatment, trabes type of invasive front and depth of invasion greater than 4 mm were confirmed to be independent predictors of TSCC cervical lymph node relapse.

In the study, the growth pattern of TSCC invasive front was classified as crumb (islet-infiltrating cells with wide fronts of invasion) or trabes (thin infiltrating cords). A third pattern with individual infiltrating cells has also been reported previously in literature [7]. However, such growth pattern was not observed in our samples. As early as in 1984, Yamamoto et al. presented that oral cavity SCC with cord-like type (i.e. trabes) of invasive front showed a higher risk of metastasis [9], which is further confirmed with the results shown our research. Concerning the depth of tumor invasion, our results were in accordance with previous studies, which proposed a cut-off value at 5 mm of tumor depth [10, 11]. Yet according to our results, metastasis was indicated in patients with the depth of invasion larger than 4 mm, in consistence with Ambrosch et al. [12]. Moreover, an anatomical invasion of tumor deeper than the intrinsic tongue muscles, paralingual and sublingual spaces, and medial and lateral extrinsic tongue muscles, was a potent indicator of cervical lymph node relapse in TSCC [13]. The effect of neurovascular invasion and histological differentiation on cervical lymph node relapse has been widely investigated in SCC [14, 15]. In our results, neither neurovascular invasion nor histological differentiation was confirmed as independent predictors of cervical lymph node relapse in TSCC. We also found that cervical lymph node relapse is the most important prognostic factor in SCC with the highest HR value in multivariate analysis.

In the results of the current study, we also observed that neck dissection did not influence the metastasis and prognosis of TSCC. Results were contradictive concerning the effect of neck dissection on TSCC patients. Some suggested that neck dissection significantly reduces the mortality and increases the 5-year disease-free survival [16–18]. Yet some proposed that neck dissection is not necessary until a node is detectable [19]. Currently, in the treatment of TSCC, an elective neck dissection is suggested only for patients with certain risk factors, as Fakih et al. proposed in their research [20]. Hence, for patients with risk factors including trabes invasive front and depth of tumor greater than 4 mm, an elective neck dissection is suggested to improve the prognosis of these patients.

Effects of alcohol drinking and smoking on TSCC was also evaluated in this study. Drinking and smoking have been regarded as risk factors for TSCC [4]. According to our results, in univariate analysis, both smoking and drinking were correlated with cervical lymph node relapse and poor prognosis. However, the effect was not significant in multivariate analysis adjusted by gender, age, pathological features of tumor, location of tumor and treatment. Similarly, in the report of Wildt et al., tobacco smoking, but not alcohol consumption, was observed to have significant influences on the prognosis in the Cox proportional hazards regression analysis, with T-stage, N-stage, clinical stage, tumor size and gender of patients as adjusted variables [21]. Moreover, it was reported that the alcohol consumption and smoking were related to the location of SCC. Tobacco smoking was correlated with a higher risk of malignancy in the retromolar area and floor of the mouth, while more SCC in the floor of mouth and tongue was observed in patients with habit of drinking [22]. Yet in our study, we failed to observe a similar result.

## Conclusions

In this cohort study, 141 TSCC patients were enrolled for the analysis of the clinicopathological features, cervical lymph node relapse and TSCC prognosis. In multivariate analysis, we identified the trabes growth pattern in the invasive front and depth of invasion larger than 4 mm as risk factors for cervical lymph node relapse. Concerning TSCC prognosis, only elder age group and trabes growth pattern of invasive front were considered as indicators for poor prognosis. Hence we suggested that preventive therapy for the cervical lymph node relapse, such as elective neck dissection, should be performed on patients with trabes growth pattern in the invasive front and depth of tumor invasion greater than 4 mm.

## Data Availability

The datasets used and analyzed during the current study are available from the corresponding author on reasonable request.

## Abbreviations

CI: Confidence interval
DFS: The Disease free survival
DSS: The Disease specific survival
HR: Hazard ratio
OR: Odd ratio
SCC: Squamous cell carcinoma
TSCC: Tongue squamous cell carcinoma
